# Supplementary material for: An analysis of structural relationship among achievement motive on social participation, purpose in life, and role expectations among community dwelling elderly attending day services
Source: PeerJ. 2016 Jan 28;4:e1655. doi: 10.7717/peerj.1655 (PMC4734058; doi:10.7717/peerj.1655)
Supplement: Appendix S1 [file peerj-04-1655-s002.docx]

**Appendix S1: Scale for Achievement Motive in Rehabilitation (SAMR)**

| Items of SAMR | strongly agree | almost agree | little agree | neither agree nor disagree | little disagree | almost disagree | strongly disagree |
| --- | --- | --- | --- | --- | --- | --- | --- |
|  |  |  |  |  |  |  |  |
|  |  |  |  |  |  |  |  |
| 1) I think that I can overcome any difficulties to achieve my goal. | 7 | 6 | 5 | 4 | 3 | 2 | 1 |
|  |  |  |  |  |  |  |  |
| 2) I like planning ingenious tactics. | 7 | 6 | 5 | 4 | 3 | 2 | 1 |
|  |  |  |  |  |  |  |  |
| 3) I do not accept to cease making an effort until I am satisfied. | 7 | 6 | 5 | 4 | 3 | 2 | 1 |
|  |  |  |  |  |  |  |  |
| 4) I think that I make more efforts than others. | 7 | 6 | 5 | 4 | 3 | 2 | 1 |
|  |  |  |  |  |  |  |  |
| 5) I do not spare any effort to recover earlier than others. | 7 | 6 | 5 | 4 | 3 | 2 | 1 |
|  |  |  |  |  |  |  |  |
| 6) I want to work on a goal accepted by people around me. | 7 | 6 | 5 | 4 | 3 | 2 | 1 |
|  |  |  |  |  |  |  |  |
| 7) I want to choose the rehabilitation that satisfies me most. | 7 | 6 | 5 | 4 | 3 | 2 | 1 |
|  |  |  |  |  |  |  |  |
| 8) I think that it is important to rehabilitate by myself. | 7 | 6 | 5 | 4 | 3 | 2 | 1 |
|  |  |  |  |  |  |  |  |
| 9) I want to work on rehabilitation that experts recommend is most effective. | 7 | 6 | 5 | 4 | 3 | 2 | 1 |
|  |  |  |  |  |  |  |  |
| 10) I want to give top priority to the rehabilitation that is most suitable for me. | 7 | 6 | 5 | 4 | 3 | 2 | 1 |
